# Protocol for a naturalistic study of residents living in an agriculturally-integrated (“agrihood”) neighborhood

**DOI:** 10.3389/fpubh.2025.1679602

**Published:** 2025-11-20

**Authors:** Jay E. Maddock, Emma C. Lewis, Deyaun L. Villarreal, Alexandra L. MacMillan Uribe, Kathryn M. Janda-Thomte, Gang Han, Charles R. Hall, Rodney X. Sturdivant, Megan S. Patterson, Meredith L. Graham, Rebecca A. Seguin-Fowler, M. Renée Umstattd Meyer

**Affiliations:** 1 Department of Environmental and Occupational Health, School of Public Health, Texas A&M University System, College Station, TX, United States; 2 Institute for Advancing Health Through Agriculture (IHA), Texas A&M AgriLife, Texas A&M University System, Dallas, TX, United States; 3 Department of Public Health, Robbins College of Health and Human Sciences, Baylor University, Waco, TX, United States; 4 Department of Epidemiology and Biostatistics, School of Public Health, Texas A&M Health Science Center, Texas A&M University System, College Station, TX, United States; 5 Department of Horticultural Sciences, College of Agriculture and Life Sciences, Texas A&M University System, College Station, TX, United States; 6 Department of Statistical Science, College of Arts and Sciences, Baylor University, Waco, TX, United States; 7 Department of Health Behavior, School of Public Health, Texas A&M University System, College Station, TX, United States; 8 Institute for Advancing Health Through Agriculture (IHA), Texas A&M AgriLife, Texas A&M University System, College Station, TX, United States

**Keywords:** agrihood, built environment, community health, nutrition, diet, physical activity, mixed methods, protocol

## Abstract

**Background:**

Poor nutrition and inadequate physical activity are key contributors to rising chronic disease rates across the United States. It is well-documented that neighborhood built environments play an important role in shaping these modifiable health behaviors. Agriculturally-integrated neighborhoods (“agrihoods”) offer a relatively new and promising approach to health-promoting residential design and development. Centered around a working farm, agrihoods are designed to connect residents with fresh foods and outdoor spaces that encourage physical activity. However, no rigorous or longitudinal evaluations of their impact on resident health have been conducted to date. We detail the protocol for a naturalistic study that aims to (1) assess short-term changes in dietary intake, physical activity, cardiometabolic health indicators, and social connectedness among agrihood and matched comparison residents; (2) document time use with and preferences for agrihood design features; and (3) examine agrihood economic benefits.

**Methods:**

This mixed-methods, quasi-experimental study will last approximately 6 months including assessment at three timepoints (baseline, 3 months, 6 months). Agrihood participants will be new adult residents in an agrihood residential development (Richmond, TX), while comparison participants will be adults currently residing in a nearby residential development (Katy, TX). All participants will complete three 30-min online surveys, including items to produce geospatial data; agrihood participants will complete two additional 30-min in-person health assessments and three timepoints of accelerometer wear on 7 consecutive days, with a subsample completing three 30-min online dietary recalls. Data will be collected by trained staff, and Difference-in-Differences (DiD) generalized linear mixed-effects models will examine longitudinal change and its interaction with participant groups. The economic analysis will account for break-even time, farmer compensation, maintenance costs, and public incentive programs.

**Discussion:**

This will be the first study to examine a longitudinal cohort of new agrihood residents compared to a master planned residential development to understand the use and health impacts of agrihood living. Our data-driven design, including biological data collection, device-captured physical activity, and validated self-report measures, can pave the way for future research, policy and philanthropic initiatives to adapt and scale models for developments that promote environmental and economic growth, and improve human health and wellbeing.

**Clinical trial registration:**

https://clinicaltrials.gov/study/NCT06950775, Identifier NCT06950775.

## Introduction

Poor nutrition and inadequate physical activity are prevalent in many communities across the United States, contributing to rising rates of chronic diseases such as obesity, diabetes, heart disease, and certain cancers (1–3). Consuming a healthy diet and being physically active are not only important for preventing these health conditions but also for managing and, in some cases, improving them (1). While socioeconomic disparities significantly influence access to healthy food and safe places to be active, a range of societal, environmental, and policy factors shape modifiable health behaviors across all socioeconomic groups (4). In fact, neighborhood built environments have been linked to outdoor activity and diet quality as well as social connection (5–10), sometimes regardless of socioeconomic status (11), pointing to an opportunity to improve health on a broader scale through the intentional design of neighborhoods that foster walkability, healthy food retail and agriculture, and social interaction.

Over the last decade, agriculturally-integrated neighborhoods called “agrihoods” have risen in popularity and present a promising avenue for positioning health at the center of land-use development (11). While there are no established definitions or required components to be considered an agrihood, these neighborhoods typically consist of residences ranging from apartments and condos to attached and single-family homes constructed around a working farm. There is often particular emphasis placed on environmental sustainability (i.e., regenerative farming practices, reduced personal vehicle use and food transportation needs) and community engagement (i.e., community-led gardens and farmers markets, educational and recreational events). Agrihoods can also help support local economies through job creation for farming, food production, and community-based enterprises (12). Property values in agrihoods tend to be higher due to the unique appeal of living close to green spaces and farms, attracting buyers seeking a healthier, eco-friendly lifestyle (13, 14). Additionally, these communities can boost tourism and local commerce by hosting farm-to-table events, farmers’ markets, and educational programs, creating a ripple effect of economic growth and resilience throughout the region (12, 15, 16).

Currently, it is believed that more than 200 agrihoods are planned or developed in the United States, spanning nearly 30 states. Despite this, a recent review by our research team found limited peer-reviewed published literature on agrihoods, and none that rigorously or longitudinally evaluate their impact on health (17). Impact evaluations, especially those using validated measures, are needed to better understand agrihood characteristics, community use of and engagement with resources and agriculture by agrihood residents, changes to agrihood residents’ health behaviors, and community involvement in the design and maintenance of agrihood environmental features and agricultural production (17). Doing so could help inform the design of replicable agrihood models that can be tailored to meet the needs of various populations and settings.

Therefore, we detail the protocol for a naturalistic study, referred to as the *Neighborhood & Health Study*, which aims to assess the impact of relocating to an agrihood on health and economic outcomes among residents of a newly developed agrihood neighborhood in Fort Bend County, Texas, compared to a geographically and socio-demographically matched non-agriculturally-integrated, master-planned neighborhood, located 23 miles away (by car) in neighboring Harris County, Texas. Given the need for evaluations of agrihood impacts over time and in various settings, we present our scalable methodology for (1) assessing short-term changes in dietary intake, physical activity, cardiometabolic health indicators, and social connectedness among agrihood residents compared to those of a matched master-planned neighborhood that is not agriculturally-integrated; (2) documenting resource utilization and time use with and preferences for built and nature design features of the agrihood; and (3) examining economic benefits of the agrihood from resident and developer perspectives.

## Materials and methods

### Design and setting

The *Neighborhood & Health Study* employs a quasi-experimental prospective case–control mixed-methods approach. This entails assessing new residents of the agrihood and a comparison group over a period of 6 months (subject to a + 3-month adjustment based on actual move-in date).

The study is primarily situated at Indigo, a new 235-acre, planned 750-home agrihood located in Richmond, Texas (Fort Bend County), about 28 miles southwest of downtown Houston (18). Its developer, Meristem Communities Development Firm, is a Houston-based real estate development firm committed to creating Places for People™ which they envision as being inclusive public spaces (19). Meristem emphasizes resiliency and human-centric design by mindfully and sustainably creating places that connect land, natural systems, and people. For this reason, among others (e.g., proximity to the research team, a development timeline that aligns with the research project timeline, and Meristem’s willingness to partner), Indigo was selected for this study.

The agrihood comprises 42 acres of farm and pasture, over half of the community dedicated to open space, a 12-acre mixed-use town center with a walkable main street design and mixed retail/businesses, a 25-acre lake, integrated trails, “the Filling Station” (welcome center, café, gathering place, farm stand), vehicle-free community areas focused on nature, and spaces for residents to interact and play between rows of houses where there would traditionally be a street. Agriculture in the agrihood will be managed by Agmenity (20), a leader in the local and national agrihood movement, and will include crop fields, orchards, pasture for livestock, year-round production of seasonal produce, farm stand, farm club, agricultural education opportunities, programming, volunteer opportunities, and pastured poultry and egg production. The agrihood was designed to provide a foundational connection to agriculture and a people-first approach that fosters community connection and overall health, while being committed to environmental stewardship.

Set to be completed in December 2026, the agrihood will include a diverse portfolio of home prices ranging from the upper $200,000’s to over $500,000 (800–3,100 sq. ft.). Median home values were $435,099 for the agrihood’s zip code in April 2025 (21, 22), demonstrating the agrihood’s commitment to providing affordable options to a diverse range of buyers in this area. Sales for homes began in March 2024 and initial move-in dates are scheduled for fall 2025. Richmond is a diverse, growing city with a racial and ethnic composition of 45.9% Non-Hispanic White, 48.5% Hispanic/Latino, 26.6% Black, and 13.8% Asian (23, 24).

The comparison community is located in Katy, Texas (Harris County), about 23-miles from the agrihood and 36-miles northwest of downtown Houston (25). The comparison neighborhood is a master-planned, non-agriculturally focused development with an estimated 2,824 single-family homes built since 2016 with build-out completion plans set for 2029. It was selected by the research team for its comparable home prices (resale values range between $419,000–$700,000 and new homes start in the $300,000’s) (21, 22) and square footage (83% 1,400–3,000 sq. ft., n = 2,011), zoning (26), and mixture of housing styles and proximity to mixed-use retail, as the agrihood. The comparison neighborhood differs from the agrihood in that its mixed-use retail is more car-dependent and not located within the community, and it is not agriculturally-integrated. However, the comparison neighborhood does have a small community garden containing 13 rentable plots, parks, connecting sidewalks, two amenity centers with physical activity options (e.g., pool, fitness center), ponds, and a prairie-land walking path. Demographically, Katy has a racial and ethnic composition of 57.4% Non-Hispanic White, 30.1% Hispanic/Latino, 12.2% Black, and 7.0% Asian (23, 24).

### Study aims and approaches

The specific aims for the *Neighborhood & Health Study* are to: (1) assess short-term changes in dietary intake, physical activity, cardiometabolic health indicators, and social connectedness among Texas residents living in the agrihood compared to non-agriculturally-integrated, master-planned neighborhood; (2) document time use with features of the agrihood and assess preferences for built and nature design features to provide resident-driven data for future developments; and (3) examine the resident- and community-level economic benefits of the agrihood.

The study design and selection of measures are guided by the socioecological model, which recognizes that health behaviors are influenced by interacting individual (e.g., diet, physical activity, stress), interpersonal (e.g., social connection), organizational (e.g., community programs), and environmental (e.g., built and natural design features) factors (27). This framework provides a foundation for examining how agrihood design features may affect resident health and wellbeing.

To conduct Aim 1, we will recruit two longitudinal cohorts, one of new agrihood residents (*N* = 175), and one of current comparison neighborhood residents (*N* = 175). Agrihood participants completing the baseline assessment will receive $100, those wearing an accelerometer and completing a mid-point survey will receive $150, and those completing the 6-month follow-up will receive $100. Comparison participants will only complete surveys and will receive $50 for the completion of a survey at each timepoint. Physical assessments will be conducted among agrihood participants by trained clinical staff in Mobile Health Assessment Centers (MHACs), which are mobile clinic spaces specifically designed for biometric data collection. MHAC measures will include blood pressure, resting heart rate, hemoglobin A1c (HbA1c), lipids, dermal carotenoids, weight and height. Seven-day accelerometer data collection will occur at baseline, mid-point, and 6-month follow-up to measure 24-h movement behavior (physical activity, sedentary time, and sleep). Geospatial data regarding access to food and physical activity resources, and resource utilization, will be collected as part of each survey timepoint. Locations of food and physical activity resources located within a 5-mile radius of the agrihood and comparison neighborhoods will be obtained via Google Distances API and mapped using ArcGIS. This will help us understand the food and physical activity resource context that these communities are located in. However, just because a resource is located proximally to a neighborhood, does not mean it is utilized (28). Therefore, we also will be asking questions to participants regarding their food acquisition and physical activity behaviors to understand how participants engage in the food and physical activity built environment. These questions include addresses of resources used, frequency of use, and time spent at resources. Survey measures for both agrihood and comparison participants include the Mediterranean Eating Pattern for Americans Screener (MEPA-16) dietary assessment (29), American Heart Association (AHA) Life’s Essential 8 (30), Perceived Stress Scale (31), and Brief Sense of Community Scale (32). The total time commitment for participants at each time point will last approximately 90 min, not including accelerometer wear time. In addition, a subsample of agrihood residents (*n* = 20) will be asked to provide an online 24-h dietary recall. See Table 1 for a detailed description of each outcome measure.

**Table 1:** Aim 1 outcome measures and their descriptions.

Measure	Description
Blood pressure	Measured in units of millimeters of mercury (mmHg) with an automated Omron sphygmomanometer. Blood pressure measurements will be repeated twice to ensure accuracy. If more than 10 mmHg difference between two measurements occurs, an additional measurement will occur. All measurements will be recorded in the study data collection form, and an average of those measures will be used in data analysis.
Heart rate	Measured in units of beats per minute (BPM) with an automated Omron sphygmomanometer concurrently with blood pressure. Heart rate measurements will be repeated twice to ensure accuracy. If more than two BPM difference between two measurements occurs, an additional measurement will be taken. All measurements will be recorded in the study data collection form, and an average of those measures will be used in the data analysis.
Hemoglobin A1c	A drop of blood will be collected using a finger prick. The drop of blood will be absorbed into a test strip that will be inserted into a small portable machine (A1CNow) that will measure the glucose in the blood. Hemoglobin A1c is measured in milligrams (mg) per deciliter (dL) of blood and the measure will be recorded in the study data collection form.
Lipids	A drop of blood will be collected using a finger prick. The drop of blood will be absorbed into a test strip that will be inserted into a small portable machine (CardioChek Plus) that will measure the lipids in their blood. Triglycerides will be measured in milligrams (mg) of triglycerides per deciliter (dL) of blood. Low-density and high-density lipoprotein (LDL/HDL) cholesterol will be measured in milligrams of LDL/HDL cholesterol per deciliter (dL) of blood. Total Cholesterol is measured in milligrams (mg) of cholesterol per deciliter (dL) of blood. All lipids will be measured using CardioChek Plus point-of-care analyzer and then recorded in the study data collection form.
Dermal carotenoids	Measured using the Veggie Meter®, which will scan the participants’ finger using light technology to measure the carotenoids and produce a score ranging from 0 to 800 (47). The higher the score, the more carotenoids are in a person’s body, indicating greater regular consumption of fruits and vegetables. An average of three consecutive measurements will be taken using the “Average of 3 Scans” mode (48). All measurements will be recorded in the study data collection form, and an average of the measures will be used in data analysis.
Weight	Recorded in kilograms. Weight measurements will be repeated to ensure accuracy. If more than a 0.1 kg difference between the two measurements occurs, a third measurement will be taken. All measurements will be recorded in the study data collection form, and an average of the measures will be used in data analysis.
Height	Measured in centimeters. Height measurement will be repeated to ensure accuracy. If more than 0.25 cm difference between two measurements occurs, an additional measurement will be taken until two consecutive measurements within 0.25 cm occur. All measurements will be recorded in the study data collection form and an average of those measures will be used in data analysis.
Online survey	The online survey will be hosted on Qualtrics (Provo, UT, USA, https://www.qualtrics.com) and is expected to take 30 min to complete. The survey assesses sociodemographics, the community food environment, fruit and vegetable consumption, stress, mood, gardening, general health, sense of community, time spent in green spaces, and physical activity. The survey will contain items from the following measures: Community Food Environment - Store Access and Shopping Behaviors (NEMS) (49), National Cancer Institute (NCI) Fruit and Vegetable Screener (50), Perceived Stress Scale (31), Gardening and Local Food – modified survey (51), Life’s Essential 8 Score (30), MEPA-16 (29), Social Connectedness and Sense of Community (52), adapted Nature Surveys (53, 54), International Physical Activity Questionnaire – Short (55), Positive and Negative Affect Scale (56), Neighborhood Social Cohesion Scale (57), Transportation Use Questions, Food and Physical Activity Resource Utilization Questions (28), UCLA Social Isolation and Loneliness Scale (58), QualityMetric’s Mean Opinion Score (MOS) Short Form (59), and sociodemographic items.
Accelerometer	We will utilize ActiGraph accelerometers (Ametris), model wGT3X-BT (https://ametris.com/actigraph-wgt3x-bt) with a wrist strap. Participants will be asked to wear the device around their wrist on their non-dominant wrist slightly above the wrist bone, like a watch. Participants will be asked to wear the device for close to 24 h each day for 7 consecutive days, including during sleep. If participants need to remove it for any reason, they will be asked to record the exact times in their log that they took the accelerometer off and put it back on. Participants will also be asked to note the time they went to sleep and the time they woke up on a paper accelerometer log.
Dietary recall (subsample)	For a subset of agrihood participants, we will utilize the National Cancer Institute (NCI) Automated Self-Administered 24-Hour Dietary Assessment Tool (ASA24) for dietary data collected via the dietary recall (https://asa24.nci.nih.gov/) (60). Through this online tool, we will ask participants to report all the foods and drinks they consumed in the last 24 h. Once they begin the dietary recall, they will have until midnight to finish entering all of the foods and drinks they consumed in the last 24 h.

To conduct Aim 2, we will recruit a subsample of adult residents living in the agrihood (*n* = 48) from the longitudinal cohort to attend a focus group and complete a time-use study. We will conduct a total of four focus groups with 12 people per group, led by trained research staff and lasting approximately 1 hour each. Topics will cover perceived positive and negative changes from living in the agrihood, consumer preferences for agrihood features and programming, including types of produce grown, ability to work on the farm, and aspects of the agrihood’s market (the Filling Station). Focus group participants will then be asked to complete a one-week time-use study. This will include once daily self-reporting via Qualtrics on neighborhood activities such as working on the farm, using the walking trails, attending events hosted at the agrihood, spending time in their home, and participating in external activities like driving, work or school, errands, and leisure activities. Focus groups participants will receive a $100 incentive for their time.

Finally, Aim 3 will be conducted as part of the agrihood resident focus groups, during which participants will be asked their reasons for moving to the agrihood, if they actively participate in the agrihood’s agricultural offerings, sales, and programs, their level of farm volunteering, and neighborhood satisfaction and place attachment. Economic benefits associated with the agrihood benefits explored in Aims 1 and 2 will be monetized. Concurrently, additional data will be acquired from the developer using a survey with items regarding break-even time, farmer compensation, maintenance costs, and public incentive programs such as open space tax credits, easements, and grants. The survey will be developed, conducted, and analyzed by a trained agricultural economist with expertise in financial analysis on the study team. Taken together, these resident- and community-level insights will provide the best current evidence base for how agrihoods create, capture, or redistribute economic value—through property-price premiums, avoided amenity costs, conservation tax policy, and diversified farm revenue.

### Participants

#### Recruitment

All recruitment activities will be overseen by trained research staff. Participants who express interest will complete a Qualtrics online screener to ascertain that they meet the inclusion criteria (described below). Interested individuals will complete the screener either independently or guided by a research staff member via phone or in person at a recruitment event. Once deemed eligible, participants will provide informed consent prior to engaging in study activities.

##### Agrihood participants

Agrihood participants, including the longitudinal cohort and focus groups, will be recruited using existing Homeowner’s Association marketing efforts, including flyers sent when homes are purchased, community fairs, front door hang tags, as well as word-of-mouth, informational presentations, phone calls, emails, website promotion, and flyers placed in community locations nearby Indigo.

##### Comparison participants

Comparison participants will be recruited using existing neighborhood marketing efforts, community fairs, front door hang tags, word-of-mouth, phone calls, website promotion, and flyers placed in community locations near the neighborhood.

#### Inclusion criteria

All participants will be adults, some of whom may be from the same household, and pregnant women may also be included. Specific inclusion criteria are below.

##### Agrihood participants

Inclusion criteria for agrihood participants are: (1) being 18 years or older; and (2) living in the agrihood as their primary or permanent residence for less than 3 months (cohort) or at least 4 months (focus group) at the time of recruitment.

##### Comparison participants

Inclusion criteria for comparison participants are: (1) being 18 years or older; and (2) currently living in the comparison neighborhood as their primary or permanent residence at the time of recruitment.

#### Sample size

**Figure 1:**
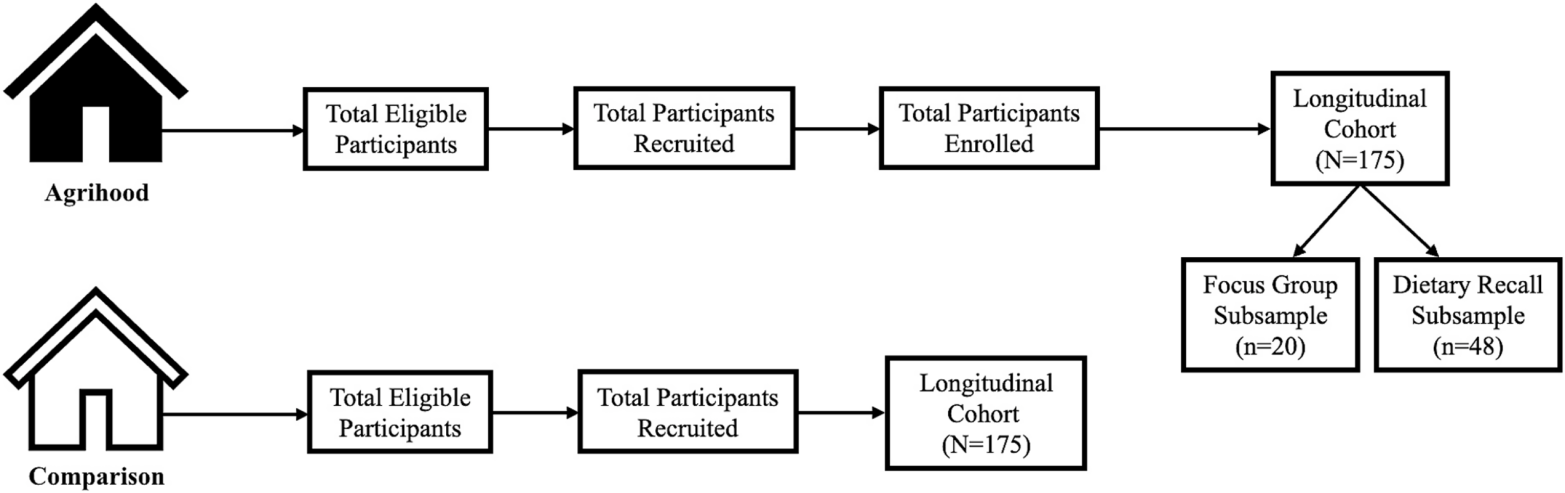
Flow and organization of participants.

A total of 175 agrihood participants and 175 comparison participants will be recruited (*N* = 350). See Figure 1 for the flow and organization of participants.

##### Sample size estimation

Power was computed for a natural experiment evaluation (NEE) using Difference-in-Difference (DiD) analysis. For Aim 1, we calculated sample size for a Type I error rate alpha of 0.05, power of 0.8 for comparing the means between two groups considering physical activity outcomes of moderate-to-vigorous physical activity (MVPA); light, moderate, and vigorous physical activity (LMVPA); and time spent in sedentary behavior. Prior research has shown observed effects of moving to an activity-friendly community ranging between 10.4–10.9 min/day of MVPA, indicating small effect sizes of 0.40–0.42 (Cohen’s D) (7). We conservatively powered this study to detect a smaller effect size (Cohen’s D = 0.35). We estimate a sample size of 129 per group will provide adequate power to detect an effect size of Cohen’s D = 0.35 for all physical activity outcomes. The effect size of 0.35 is a conservative estimate based on current literature, equating to minimal detectable differences between 7.9 and 9.6 min/day for MVPA using estimates of standard deviation from previous studies (7, 33–37). This sample size is also adequate for a minimal detectable difference of 13.3 min/day for LMVPA. This sample size is adequate to detect effect sizes that are small to medium for all outcomes based on calculations for other aims. Prior community sampling of an NEE examining accelerometer-measured MVPA found a 20.64% attrition rate from baseline to year 1 and a 6.63% attrition rate from year 1 to year 2 (33). Given recently reported challenges to NEE recruitment, we conservatively overestimate attrition rates, necessitating a sample size of 175 for the agrihood and comparison groups.

##### Loss to follow-up

A participant will be considered lost to follow-up if they fail to return for any of the scheduled follow-up visits, or do not complete that timepoint’s survey, and cannot be contacted by the research team by the end of the study period. In the case of loss to follow-up or withdrawal, partial data will be used in the data analysis with multiple imputation used to address missing data where appropriate. Intent-to-treat will be used to preserve the group allocation in our analysis (38).

### Data collection

The research team conducted an initial survey of demographic and health indicators, neighborhood design preferences, and willingness to pay for specific amenities among 128 prospective buyers of the agrihood between March and July 2024 (Baylor IRB #2158321–1). Findings from the survey provided a formative understanding, which helped to inform the design of the present study and served as a project pilot test for survey recruitment and enrollment procedures (39, 40).

Beginning in Spring 2025, data collection began across three time points (pre-move-in, 3 months after move-in date, and 6 months after move-in date) by trained research and clinical staff (see Table 1). Over the course of 6 months, agrihood participants will be asked to complete (1) three 30-min online surveys; (2) two 30-min in-person health assessments collecting height, weight, blood pressure reading, heart rate, dermal carotenoid scan (Veggie Meter®), HbA1c (finger prick), and lipids (finger prick); (3) three 7-day consecutive accelerometer wears; and (4) three 30-min online dietary recalls (subsample). Comparison participants will be asked to complete three 30-min online surveys at 0 months, 3 months, and 6 months from recruitment.

The focus groups are planned to occur starting in the Fall of 2025. The accompanying one-week time-use study, entailing once-daily self-reports, will begin the following day after each of the four focus group sessions. The gathering of economic data will occur during focus group discussions and concurrently with the agrihood developer as homes continue to be built and residents move into the new units. The developer survey will be designed to capture the full economic arc of an agrihood project—from conception to financial stabilization—by prompting developers to narrate each phase of development (e.g., project origins and strategic intent, land acquisition and zoning, entitlement and permitting processes, capital investment structures, farm-related costs and revenue projections, marketing, sales absorption rates, and plans for long-term stewardship).

### Data management

Data including the screening survey, online survey, time-use survey, accelerometer log, and in-person health assessment data collection form will be entered into Qualtrics, a secure data capture system. The data system includes password protection and internal quality checks, such as automatic range checks, to identify data that appears inconsistent, incomplete, or inaccurate. Paper copies of any forms and logs will be stored in a safe cabinet with a lock located at a secure research center. For data captured as part of the online dietary recall, we will utilize the ASA24 system, which comprises a secure database where responses are encrypted during transmission using Secure Socket Layer (SSL) technology.

### Data analysis

For Aim 1, we will compare the changes in physical activity outcomes (moderate to vigorous physical activity [MVPA], light, moderate, and vigorous physical activity [LMVPA], walking, sedentary behavior) between the agrihood and comparison participant groups across three time points (Agrihood: pre-move-in, 3 months after move-in date, 6 months after move-in date; Comparison: 0, 3, 6 months) using DiD analysis (linear mixed-effects longitudinal models). Regression models will include the group, time period, and the group by time period interaction terms. Random cluster effects will be included to account for correlations between participants in the same neighborhood and *a priori* covariates of age, sex, and educational attainment. Random effects for the participants will be included to account for correlation in observations. To address multicollinearity, we will assess variance inflation factors (VIF > 5) and pairwise correlations (|r| > 0.70); collinear variables will be excluded, combined, or tested in sensitivity analyses to avoid attenuation of the group × time effect. Confounders showing baseline imbalance (standardized mean difference > 0.10) will be prioritized for inclusion. Restricted maximum likelihood will be used for complete case analysis to incorporate all available data, and intention-to-treat analysis will include all participants included at baseline. Multiple imputation will be used to estimate missing data points and standard errors. Imputations will follow standardized procedures, including auxiliary variables, and will follow hierarchical approaches. We will examine the potential for missingness of data to not be at random by comparing baseline characteristics of participants who have completed physical activity data with those who do not. If there are differences by age, education, race, or self-reported perceived health, any variables that differ will be used as an auxiliary variable in multiple imputation. Fraction of missing information will be used to measure level of uncertainty about the values imputed for missing values. We will use a number of imputations that satisfy the recommendation to have the number of imputations (at least) equal the highest fraction of missing information (FMI) percentage. Using the geographic information system (GIS) data and resulting maps, for physical activity resources we will: (1) count the number of resources located within the 5-mile network buffer using ArcGIS; (2) measure the distances to the closest resources for different types of resources (e.g., closest park or trail) using ArcGIS and Google Distances API; (3) assess how these counts and distances change over time; and (4) assess changes in time spent in transportation to go to resources. Specifically, we will compare the changes in number of physical activity resources, distance (in miles) to closest objectively measured resources, and distance (in miles) to utilized resources and associated transportation-related time spent between the groups across 3 time points (Agrihood: 0, 3 months after move-in date, 6 months after move-in date; Comparison: 0, 3, 6 months) using DiD analysis, and assess if any of these variables are associated with device-measured physical activity outcomes using linear mixed-effects multilevel models. All statistical analysis will be conducted using SAS and/or R software. We will also generate maps showing changing patterns in physical activity resource utilization in ArcMap. This two-pronged approach of mapping and analyzing objective physical activity environments as well as physical activity resource utilization and time use behaviors are important to better understand how the built environment in these communities are or are not conducive to physical activity, as well as how participants are (or are not) engaging in the resources designed to promote physical activity.

We will use the same strategy above to compare changes in food sourcing and dietary intake between groups across 3 time points (Agrihood: 0, 3 months after move-in date, 6 months after move-in date; Comparison: 0, 3, 6 months) using DiD analysis. MEPA-16 score will serve as the main dietary intake outcome for all participants, while ASA-24 nutrient and food group data will be examined among the Agrihood subsample. We will also compare the changes in number of food resources (by type), distance (in miles) to closest objectively measured food resources, and distance (in miles) to utilized food resources and associated changes in time spent in transportation to food resources between the groups across three time points (Agrihood: 0, 3 months after move-in date, 6 months after move-in date; Comparison: 0, 3, 6 months) using DiD analysis. We will assess if any of these variables are associated with dietary intake using linear mixed-effects multilevel models, with MEPA-16 score as the main outcome of interest. We will generate maps showing changing patterns in food resource utilization in ArcMap. Similar to the physical activity built environment, this approach of mapping and analyzing objective food resource environments, food resource utilization, and time use behaviors can help better understand how the built environment in these communities are or are not conducive to improving food access, connection to agriculture, and healthy eating, as well as how participants are (or are not) engaging in the resources designed to promote connection to agriculture and healthy eating.

In addition, we will explore the impact of social connection and loneliness on physical activity and healthy eating using the same DiD analyses but including social connection and loneliness terms into each of the regression models. We will analyze the survey data for loneliness and social connection over time using random effects models to assess change, and determine factors related to the changes. Additionally, we will explore mediation models using the changes in loneliness/connection scores as mediators for physical activity and dietary intake changes, using methods for analysis of mediation in generalized linear models.

For Aim 2, as described for Aim 1, we will use DiD analysis to explore longitudinal changes for residential time use and engagement outcomes (i.e., assess consumer preferences for built and nature design features). Self-report commuting time and time spent outside of the agrihood will also be measured to create dose measures for overall time spent in the neighborhood. Comparison participants will be asked about the time they spend engaging in features of their neighborhoods to allow for comparisons.

For Aim 3, an exploratory economic benefits analysis will be conducted using monetized data collected from participants (as part of Aims 1 and 2) accompanied by financial data and survey measures gathered from the developer. In regard to analysis of the resident data, descriptive analysis of demographics will be coupled with a thematic analysis of responses to open-ended questions asking residents to rate their perceived importance of the working farm, open-space views, trail networks, school quality, and other standard amenities, and price premiums they believe they will pay for these. This analysis will also examine estimated monthly expenditures on farm products compared to spending at conventional grocery stores or farmers’ markets; hypothetical willingness-to-pay to retain farm amenities; anticipated influence of the farm on diet, outdoor activity, sense of community, and children’s environmental awareness; understanding of any farm-related HOA fees and decision-making processes; and perceived risks and desires. Findings will yield comparable metrics for future cross-case analyses across agrihoods, linking residents’ expectations and preferences based on their open-ended responses, to community-level economic outcomes gathered from the developer survey.

## Discussion

Consuming a healthy diet and being physically active are crucial for preventing, managing, and improving chronic diseases, yet many communities lack access to fresh foods or safe spaces for physical activity. Neighborhood built environments, particularly those that integrate agricultural production, can be intentionally designed to facilitate walkability, healthy food purchasing, and social interaction. With more than 200 planned or developed agrihoods across the country, a need has been identified for rigorous, longitudinal evaluations to better understand the impact these neighborhoods have on residents’ health and surrounding economies. The present paper details our scalable protocol for a naturalistic study in Fort Bend County, Texas.

By combining two longitudinal cohorts of matched residents, we will address three specific aims with the following expected outcomes based on our team’s experience and formative research. First, from Aim 1 (assessing short-term changes in dietary intake, physical activity, cardiometabolic health indicators, and social connectedness), we expect to demonstrate how physical activity and sedentary behaviors and locations change for adults who relocate to the agrihood compared to adults who do not. We will examine the impact of relocating to the agrihood on MVPA, LMVPA, walking, and sedentary behavior, and expect to find changes in physical activity resource utilization. We expect to explain how living in the agrihood impacts social connection and determine how connection relates to physical activity and healthy eating among participants across time. With this newly generated knowledge, we will be able to more fully explain the dynamic relationship between social connection, built environment, and chronic disease prevention. From exploratory Aim 2 (documenting time use with features of the agrihood and assessing consumer preferences for built and nature design features to provide resident-driven data for future developments), we expect to describe how residents utilize the various features of the agrihood and how much time they spend engaging in these features of their neighborhood. Finally, from exploratory Aim 3 (assessing the economic benefits of the agrihood), we expect to describe resident and developer perspectives of the cost-efficiency of living in and running an agrihood and agrihood farm model.

Our study design has several strengths, including multiple matched cohorts of residents. In addition, we propose a strong data-driven approach involving biological data collection from the mobile health assessment clinics and device-captured physical activity, along with highly valid and reliable self-report measures that capture individual and community-level preferences and perspectives. Given our multi-disciplinary team and established partnerships with the developers, we anticipate the aims will be achievable, and the results generated from this work will translate into meaningful considerations for residents of these neighborhoods. Moreover, we believe the results will allow us to understand not only how agrihoods affect health and wellbeing, but also how residents engage with them, thereby informing future models for development that are effective for improving health and are economically sustainable.

Given funding and scope of work parameters, this study also has several limitations worth noting. First, the comparison community consists of existing residents, meaning we are unable to capture their baseline prior to moving in, unlike with the agrihood participant sample. Capturing residents as they move into a new development is extremely challenging, and it was not feasible to find a comparison community that was being occupied at the same time as the agrihood. Second, due to budgetary constraints, accelerometer-based physical activity measurement and biological data collection are only feasible among participants in the agrihood community, which may reduce cross-group comparability. Third, the funding support received for this work only covers 13 months, thereby allowing for 6 months of follow-up which may be too short to observe meaningful or sustained behavioral and health changes.

Additional challenges may arise with retention from recruitment events to follow-up in-person physical assessments. The MHACs are based in Dallas, Texas, and our team has limited days for on-site biological data collection, which may create scheduling barriers. To mitigate this, participants will be offered flexible options such as completing online surveys remotely and receiving accelerometers by mail or at a designated community pick-up location (the Filling Station). Higher-than-usual attrition was anticipated and accounted for in our study design. The protocol also allows for multiple adults (e.g., married couples) within the same household to participate. This decision was made to help achieve recruitment and enrollment goals given the number of households under contract. Interclass correlations within households will be accounted for statistically in the analysis.

For cholesterol and HbA1c data, we are using finger prick sampling rather than venipuncture. Although venipuncture is often considered more precise, capillary testing has been shown to produce comparable results when performed properly and is substantially less invasive and more acceptable in community-based research settings (41–45). Self-reported survey data also introduce potential recall and social desirability bias. The economic analysis component is exploratory, drawing on developer-provided data and resident perceptions. While this approach supports early feasibility insights, it limits the ability to conduct standardized cost–benefit or return-on-investment modeling. Future work will incorporate objective financial metrics (e.g., property values, healthcare cost offsets) to strengthen policy and planning relevance.

Finally, this study is limited to one agrihood in a single region (Fort Bend County, Texas), which may restrict generalizability to other cultural, climatic, or socio-economic contexts. Despite these limitations, this pilot study represents a critical first step in establishing the feasibility, logistics, and interdisciplinary partnerships needed for more rigorous future work. The present phase provides foundational data to inform a larger, multi-site randomized controlled trial with extended follow-up, expanded objective data collection, and greater diversity among participants.

In conclusion, agrihoods present an innovative and relatively novel idea for building healthier communities. As people have reduced outdoor time and moved away from farms over the last few decades, many are now disconnected from nature and the process of growing food (46). If this study is successful, it may provide evidence of the health benefits of this type of urban design and help to shape future neighborhoods. Like all natural experiments, this research design does have some limitations. These are counterbalanced by a high level of external validity. The data collection methodologies are solid and provide short-term indications of changes in biological indicators and health behaviors. We will actively seek funding to include 12- and 24-month follow-ups, as well as to support future scale-up to larger evaluations and the development of interventions and initiatives informed by the findings from the present study.
